# The effectiveness of mobile app usage in facilitating weight loss: An observational study

**DOI:** 10.1002/osp4.757

**Published:** 2024-05-13

**Authors:** Rosemary Huntriss, Rodion Salimgaraev, Dimitri Nikogosov, John Powell, Krista A. Varady

**Affiliations:** ^1^ Simple.Life Apps, Inc. Dover Delaware USA; ^2^ Nuffield Department of Primary Care Health Sciences University of Oxford Oxford Oxfordshire UK; ^3^ Department of Kinesiology and Nutrition University of Illinois Chicago Illinois USA

**Keywords:** fasting, obesity, overweight, weight loss

## Abstract

**Aim:**

With increasing rates of global obesity and associated health issues, there is an ever‐increasing need for weight management solutions to be more accessible. Mobile applications offer accessible support systems and have the potential to offer a viable and effective weight management solution as an alternative to traditional healthcare models.

**Objective:**

To evaluate the effectiveness of the SIMPLE mobile application for time‐restricted eating in achieving weight loss (WL).

**Methods:**

User data were analyzed between January 2021 and January 2023. In‐app activity was calculated as the proportion of active days over 12, 26 and 52 weeks. A day is considered active if it contains at least one in‐app action (e.g., logging weight, food, fasting, or physical activity). Users were categorized into four in‐app activity levels: inactive (in‐app activity <33%), medium activity (33%–66%), high activity (66%–99%), and maximal activity (100%). Weight change among in‐app activity groups was assessed at 12, 26, and 52 weeks.

**Results:**

Out of 53,482 users, a positive association was found between the use of the SIMPLE app and WL. Active app users lost more weight than their less active counterparts. Active users had a median WL of 4.20%, 5.04%, and 3.86% at 12, 26, and 52 weeks, respectively. A larger percentage of active users—up to 50.26%—achieved clinically significant WL (≥5%) when compared to inactive users. A dose‐response relationship between WL and app usage was found after adjusting for gender, age, and initial Body Mass Index; a 10% increase in app activity correlated with increased WL by 0.43, 0.66 and 0.69 kg at 12, 26, and 52 weeks, respectively.

**Conclusions:**

The study demonstrates that the SIMPLE app enables effective WL directly associated with the level of app engagement. Mobile health applications offer an accessible and effective weight management solution and should be considered when supporting adults to lose weight.

## Graphical abstract

The more people use the SIMPLE app, the more weight they lose. “Home”, “Explore” and “Progress” screens of the iOS SIMPLE app, v7.7.7 (March 2024).

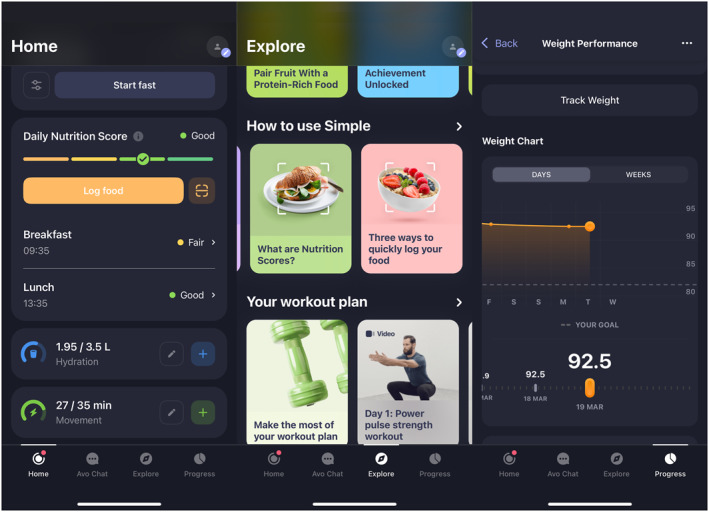

## 1 INTRODUCTION

The prevalence of overweight and obesity has risen significantly in recent decades, posing a global public health issue. Obesity contributes to early mortality^1^, ^2^ and a diminished quality of life due to various associated chronic diseases.^3^, ^4^, ^5^, ^6^ Early interventions such as nutrition, physical activity, and behavior change strategies can effectively prevent or slow down the progression of these diseases,^7^, ^8^ often as a result of weight loss (WL). One such intervention is intermittent fasting.^9^, ^10^ Although there are many types of intermittent fasting, they all share the general principle of alternating periods of fasting (abstinence or very low energy intake) with periods of eating. Time‐restricted eating (TRE) is a common intermittent fasting method that limits the number of daily hours during which a person consumes food and calorie‐containing drinks. Common TRE schedules include 12:12, 14:10, 16:8, 18:6, or 20:4, the first number guiding the fasting hours and the second guiding the duration of the eating window. TRE has been shown to result in WL for many,^11^ and the process of fasting itself may involve other physiological processes that promote overall health.^12^, ^13^, ^14^ Thus, research suggests that intermittent fasting may lower the risk of certain chronic diseases like Type 2 diabetes^15^ and heart disease,^16^ while potentially improving metabolic health^15^, ^17^, ^18^ through several mechanisms—most notably WL. In addition, intermittent fasting may also become a lifestyle habit that helps people maintain WL.^19^, ^20^

Mobile applications are an alternative support system that is often more accessible and less resource intensive than in‐person care.^21^ Existing evidence suggests that interventions delivered via mobile applications can be successful for WL.^21^, ^22^ A recent study conducted by Torres et al., (2022) demonstrated significant WL among participants with overweight and obesity using an intermittent fasting app.^23^ Similarly, Valinskas et al., (2023) found that greater engagement with an intermittent fasting app was associated with significantly greater WL in participants with obesity.^24^ However, further studies including more participants are needed to validate findings and expand the evidence related to various time periods and to individuals living with overweight.

Thus, the objective of this study was to explore the effectiveness of a mobile app in supporting adults to lose weight while examining the association between the level of app engagement and WL success.

## 2 METHODS

**FIGURE 1:**
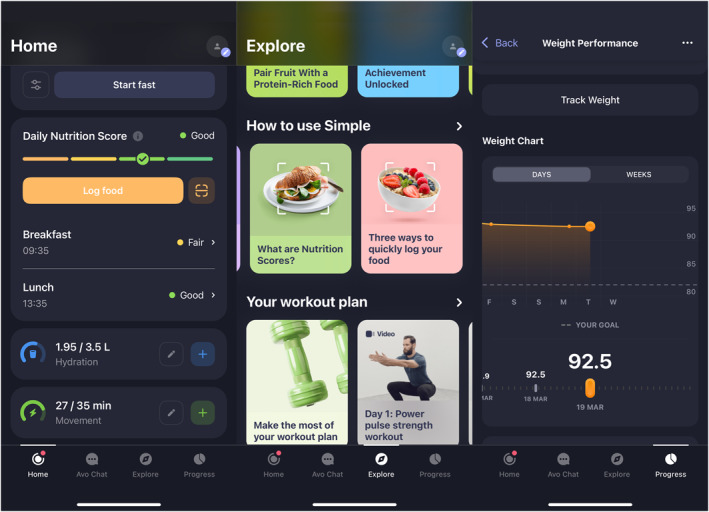
“Home”, “Explore” and “Progress” screens of the iOS SIMPLE app, v7.7.7 (March 2024).

The study utilized data from SIMPLE, a free mobile application with several features available only in a paid subscription plan, developed by Simple.Life Apps Inc. The SIMPLE app is available in most countries in a number of languages including English, French, German, Italian, Portuguese and Spanish, and aims to assist users in reaching and maintaining their WL goals by providing support on integrating TRE, nutrition and lifestyle habits. The SIMPLE app provides trackers for fasting (by tracking the start and end times of fasting periods), food, drinks, physical activity and weight, with the option to integrate data from third‐party platforms such as Apple Health, Google Fit, smart scales, and wearable devices. The application empowers users to create and sustain behavior change through reminders, an extensive library of educational articles about IF, healthy eating, workouts, etc., and recipes, personalized insights and feedback based on in‐app behaviors (Figure 1).

### 2.1 Participants

SIMPLE application users were included as a study participant if they started using the application between January 1, 2021 and January 1, 2023 and fulfilled all of the following criteria: age ≥18 and ≤70 years; baseline body weight ≥45 and ≤250 kg; height ≥150 and ≤250 cm; baseline Body Mass Index (BMI) ≥ 25 and ≤60 kg/m^2^; male or female sex; selected TRE schedule from 12:12 to 23:1 (fasting for 12–23 h, followed by an unrestricted eating window for the remaining hours of the day); recorded body weight at least 3 times during the study period (including their baseline weight); at least 12, 26 or 52 active days during 12, 26 or 52 weeks of observation, respectively; at least one weight track within 90 days after baseline weight track.

Those who were pregnant, breastfeeding, or reported an eating disorder were excluded from the SIMPLE application and therefore from the study.

Other criteria, such as weight, height and upper BMI limit were determined by the guidance of the 1st and 99th percentiles within the dataset, capturing a wide range of study participants with sufficient data to allow for meaningful analyses while removing outliers.

Missing data was not present in the dataset due to the nature of the filtration process.

### 2.2 Data collection

Data collected from the database included self‐reported gender, height and age collected when users signed up for the application, in addition to self‐reported baseline weight recorded in the app and continuous logs of food, drink, activity, fasting or weight throughout the duration of the study period.

Outliers were removed from user body weight records; any weight records that changed from the previous record by more than 5% within a 7 day period were excluded.^25^ This process of outlier filtration starts from the baseline weight record and continues until all weight records for the user are assessed.

### 2.3 Observation periods

After collecting and filtering the weight data, weight change was considered within three observation periods: 12 (the first three months of observation), 26 (the half of the first year), and 52 (the whole year) weeks, calculated as the difference between the user's baseline weight and their recorded weight closest to the end of the observation period. These time periods were chosen a priori to reflect the follow‐up periods of most interest to researchers.

An assessment period was determined within which data could be collected for that period and calculated as ±10% of the observation period in days rounded up for the start and rounded down for the end of the observation period: 12 weeks (84 days) ± 75–93 days; 26 weeks (182 days) ± 163–201 days; 52 weeks (364 days) ± 327–401 days. Users who did not enter a weight during the assessment period were not included in the analysis of the corresponding observation period.

### 2.4 Engagement with SIMPLE app

The independent variable of interest in this study was the in‐app activity of the user. A day was counted as active if at least one of the following tracking activities was manually self‐reported: weight, physical activity, fasting (a fast was started or finished), food or drink. User in‐app activity is a proportion of active days in a specific observation period. The value of in‐app activity varies between 0.0% and 100.0%, where a higher value represents a higher engagement with the app.

Due to the significant representation of the group of users with activity equal to 100%, especially in the first observation period, this group was highlighted and analyzed separately.

Users were divided into the following groups of in‐app activity, as previously described by Ingels et al. (2017)^26^: inactive (low) — proportion of active days during observation period is between ≥0 and <33%; active (medium) — ≥ 33% and <66%; active (high) — ≥ 66% and <100%; active (maximal) — 100%.Sex, baseline age, and BMI were included as additional variables. In‐app activity of a user can change between observation periods.

For the context of the interpretation of results, users are considered an Active User if they log any activity on at least 33% of the days within the observation period, falling into either the medium, high, or maximal in‐app activity groups.

### 2.5 Weight change

To estimate weight change, in each observation period, the absolute and percentage difference between the baseline and final recorded weights in the corresponding assessment period was calculated. Additionally, the proportion of users who achieved clinically significant WL (≥5% and ≥10% of body mass)^27^ in each observation period was calculated, and the proportion of users who lost weight or maintained their weight was recorded. A user was considered to have maintained their weight if their weight did not change by more than 1% from their baseline body weight.

### 2.6 Statistical analysis

The analysis was performed using Python v3.9.13,^28^ SciPy v1.9.1^29^ and statsmodels v0.13.5 libraries.^30^ Statistical significance of differences in continuous metrics between activity groups was analyzed using the Kruskal‐Wallis test, and the Chi‐squared test was used for categorical metrics.

Linear regression was used to test the relationship between user in‐app activity and WL. The following were assessed: WL in kilograms and percentage of WL. Logistic regression was used to test the relationship between user in‐app activity and achievement of ≥5% or ≥10% WL.

For each observation period, 5 models were created for each outcome of interest using the following independent variables: overall in‐app activity (tens of percent of active days during the observation period); sex (male or female); age (years in decades); initial BMI (kg/m^2^). To assess the effect of age and in‐app activity on WL, decades and percentage in tens were used for illustrative purposes.

With Bonferroni correction for multiple comparisons, the alpha level of significance was 0.00079 (7.9 × 10^−4^) for all tests performed.

### 2.7 Regression assumptions and model quality

To assess the statistical significance of each of the coefficients, a t‐statistic was used. The quality of the model was evaluated using F‐statistics and assessing multicollinearity between independent variables, analysis of the distribution of the residuals, and checking for linearity between dependent and independent variables. Additionally, skew and kurtosis metrics were used for each model to estimate the normality of the residual distribution.

Ethical approval was obtained for this retrospective study from the Independent Ethical Review Board (WCG IRB) (Study Number: 1348695) in February 2023.

## 3 RESULTS

Following the eligibility assessment and outlier detection and removal, data were available for the following number of users in each observation period: 12 weeks–36,950 users; 26 weeks–22,090 users; 52 weeks–14,240 users. The total number of unique participants in this study was 53,482. Baseline characteristics are shown in Table 1.

**TABLE 1:** Participant baseline characteristics.

Sample characteristic	12 weeks (*n* = 36,950)	26 weeks (*n* = 22,090)	52 weeks (*n* = 14,240)
Demographics			
Female	26,690 (72.23%)	15,667 (70.92%)	9791 (68.76%)
Male	10,260 (27.77%)	6423 (29.08%)	4449 (31.24%)
Age, years [mean (SD)]	43.10 (11.79)	43.17 (11.72)	43.56 (11.55)
Age, years			
18–25	2737 (7.41%)	1637 (7.41%)	968 (6.80%)
26–35	7594 (20.55%)	4462 (20.20%)	2724 (19.13%)
36–45	11,147 (30.17%)	6641 (30.06%)	4378 (30.74%)
46–55	9404 (25.45%)	5709 (25.84%)	3808 (26.74%)
56–65	5120 (13.86%)	3097 (14.02%)	2021 (14.19%)
66–70	948 (2.57%)	544 (2.46%)	341 (2.39%)
Anthropometrics			
Baseline weight, kg [median (IQR)]	88 (78.00, 102.69)	88 (78.00, 102.06)	88 (77.60, 101.65)
Baseline BMI, kg/m^2^ [median (IQR)]	31 (27.93, 35.42)	31 (27.89, 35.24)	31 (27.73, 34.88)
Baseline BMI, kg/m^2^			
25.0–29.9	15,672 (42.41%)	9547 (43.22%)	6411 (45.02%)
30.0–34.9	11,453 (31.00%)	6799 (30.78%)	4339 (30.47%)
35–39.9	5658 (15.31%)	3349 (15.16%)	2067 (14.52%)
40–59.9	4167 (11.28%)	2395 (10.84%)	1423 (9.99%)
In‐app activity, % of active days [median (IQR)]	64 (33, 96)	41 (24, 76)	29 (20, 51)
In‐app activity, % of active days			
Inactive (low)	9511 (25.74%)	8991 (40.70%)	8167 (57.35%)
Active (medium)	9462 (25.61%)	6508 (29.46%)	3628 (25.48%)
Active (high)	11,969 (32.39%)	5011 (22.68%)	1981 (13.91%)
Active (maximal)	6008 (16.26%)	1580 (7.15%)	464 (3.26%)

*Note*: Variable distributions are reported as *n* (%) unless otherwise specified.

### 3.1 In‐app activity

**FIGURE 2:**
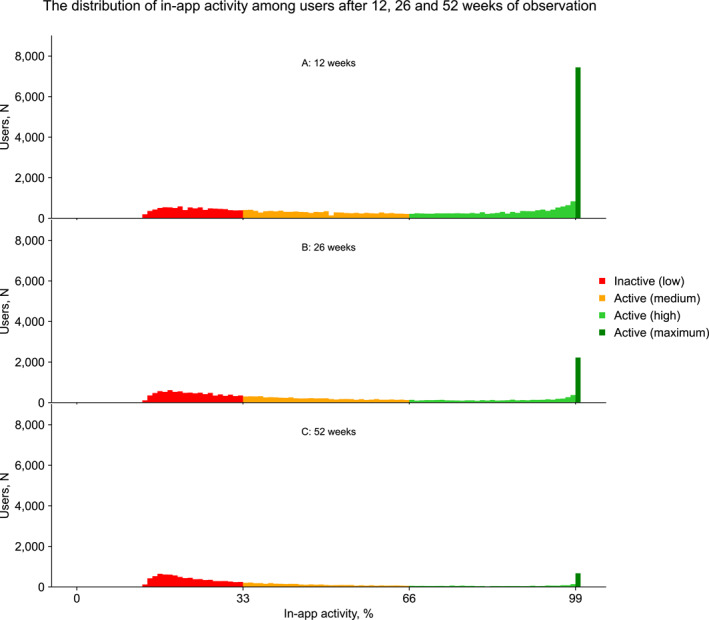
Distribution of in‐app activity among users after 12, 26 and 52 weeks of observation.

The distribution of the in‐app activity is bimodal for all three observational periods with a notable proportion of maximally active app users (Figure 2).

#### 3.1.1 Active Users

This study observed significant WL achievements among Active Users over 52 weeks. Active Users, those with medium to maximal activity levels, showed a consistent pattern of WL. The most notable finding was at the 12‐week mark, where a substantial portion of Active Users achieved a meaningful WL milestone. Specifically, over 42% of Active Users experienced at least 5% WL. This trend, along with detailed WL metrics, is elaborated in Table 2.

**TABLE 2:** Outcomes of interest for each observation period.

	Outcome of interest	Observation period		
		12 weeks	26 weeks	52 weeks
Active users	Weight loss (kg)	3.72 (1.36, 6.62)	4.53 (1.09, 8.98)	3.40 (−0.21, 8.62)
	Weight loss (%)	4.20 (1.54, 7.19)	5.04 (1.26, 9.69)	3.86 (−0.25, 9.53)
	Proportion of ≥1% weight losers (%)	79.45 (78.97, 79.93)	76.62 (75.89, 77.34)	67.89 (66.72, 69.06)
	Proportion of ≥5% weight losers (%)	42.52 (41.93, 43.10)	50.26 (49.41, 51.12)	44.20 (42.95, 45.44)
	Proportion of ≥10% weight losers (%)	11.11 (10.74, 11.48)	23.73 (23.00, 24.46)	23.50 (22.43, 24.56)
All users	Weight loss (kg)	3.10 (0.70, 6.00)	3.20 (0.08, 7.35)	2.11 (−1.27, 6.57)
	Weight loss (%)	3.52 (0.80, 6.59)	3.64 (0.07, 8.08)	2.43 (−1.42, 7.24)
	Proportion of ≥1% weight losers (%)	73.42 (72.97, 73.87)	69.01 (68.40, 69.62)	59.66 (58.86, 60.47)
	Proportion of ≥5% weight losers (%)	36.78 (36.29, 37.28)	40.98 (40.33, 41.63)	34.45 (33.69, 35.21)
	Proportion of ≥10% weight losers (%)	9.45 (9.15, 9.75)	17.91 (17.41, 18.42)	16.19 (15.59, 16.80)

*Note*: Variable distributions are reported as median (IQR for kg and %) and proportions of users achieved ≥1%, ≥5%, ≥10% weight loss (CI95).

As the study progressed to weeks 26 and 52, Active Users maintained a favorable WL trajectory although the proportions slightly fluctuated. The detailed WL figures for these periods are available in Table 2, providing a comprehensive view of the changes over time.

#### 3.1.2 All users

When considering the entire cohort, including both Active and Inactive Users, the WL outcomes at 12, 26, and 52 weeks were slightly lower compared to the Active Users group. The proportions of users achieving at least 5% WL in these timeframes are documented in Table 2.

Interestingly, the analysis of different engagement levels among Active Users revealed a clear trend. At 12 weeks, the proportions of users achieving at least 5% WL were 20.24% (95% CI: 19.43, 21.05) for inactive users and ascended to 53.15% (95% CI: 51.88, 54.41) for maximum in‐app activity users. These findings, along with the median weight losses for medium, high, and maximal active users, are detailed in Table 3.

**TABLE 3:** Outcomes of interest in different observation periods in different in‐app activity groups: inactive (low)—proportion of active days during observation period is between ≥0 and <33%; active (medium)— ≥33% and <33% and 66%;active (high) —≥66% and <66% and 100%;active (maximal)—100%.

Observation period	Outcome of interest	In‐app activity			
		Inactive (low)	Active (medium)	Active (high)	Active (maximal)
12 weeks	Absolute weight loss, kg	1.36 (−0.54, 3.90)	2.54 (0.37, 5.35)	4.17 (1.81, 7.00)	4.76 (2.36, 7.67)
	Relative weight loss, %	1.54 (−0.63, 4.26)	2.83 (0.44, 5.87)	4.64 (2.09, 7.55)	5.28 (2.75, 8.20)
	Proportion of ≥5% weight losers, %	20.24 (19.43, 21.05)	31.12 (30.19, 32.06)	46.19 (45.30, 47.09)	53.15 (51.88, 54.41)
	Proportion of ≥10% weight losers, %	4.66 (4.23, 5.08)	7.68 (7.15, 8.22)	11.95 (11.37, 12.53)	14.83 (13.93, 15.73)
26 weeks	Absolute weight loss, kg	1.70 (−0.91, 4.92)	3.30 (0.23, 7.10)	5.62 (2.09, 10.50)	6.26 (2.61, 11.07)
	Relative weight loss, %	1.90 (−1.03, 5.49)	3.71 (0.26, 7.89)	6.30 (2.36, 11.29)	7.16 (2.93, 12.09)
	Proportion of ≥5% weight losers, %	27.45 (26.53, 28.37)	40.84 (39.65, 42.04)	58.17 (56.81, 59.54)	63.99 (61.62, 66.35)
	Proportion of ≥10% weight losers, %	9.44 (8.84, 10.05)	16.86 (15.95, 17.77)	29.83 (28.57, 31.10)	32.66 (30.35, 34.97)
52 weeks	Absolute weight loss, kg	1.32 (−1.81, 5.10)	2.63 (−0.80, 7.00)	4.71 (0.59, 10.80)	6.00 (1.22, 11.57)
	Relative weight loss, %	1.47 (−2.07, 5.67)	2.93 (−0.89, 7.75)	5.66 (0.69, 11.73)	6.85 (1.56, 12.92)
	Proportion of ≥5% weight losers, %	27.78 (26.81, 28.75)	37.38 (35.80, 38.95)	53.05 (50.86, 55.25)	59.70 (55.24, 64.16)
	Proportion of ≥10% weight losers, %	10.76 (10.09, 11.43)	17.89 (16.64, 19.14)	30.64 (28.61, 32.67)	36.85 (32.46, 41.24)

*Note*: Variable distributions are reported as median (IQR for kg and %) and proportions of users achieved ≥1%, ≥5%, ≥10% weight loss (CI95). All differences in distribution between groups are statistically significant, therefore the *p*‐values are not listed.

Linear trends in WL relative to in‐app activity levels were also evident at 26 and 52 weeks, with the maximal activity group consistently showing the highest median WL. The proportions of users achieving at least a 5% WL in these groups are further detailed in Table 3.

### 3.2 Adjusted effects of different factors on weight loss

After adjustment for sex, age and baseline BMI using regression analysis, it was observed that the trend of the increase of WL with the increase of app usage was still statistically significant for all outcomes of interest in all observation periods. Among other factors, a higher baseline BMI was associated with greater WL throughout the entire observation and participants of a greater age were found to lose less weight at both the 12th and 26th weeks. Male sex was positively associated with the WL performance only during the first 12 weeks of WL. After that point, female app users tended to lose more weight (Tables 4 and 5).

**TABLE 4:** Multiple linear regression analysis results.

Observation period	Dependent variable	Independent variables							
		Gender, female		Age, decades		Baseline BMI, kg/m^2^		In‐app activity, tens %	
		*β* (95% CI)	*p*‐value	*β* (95% CI)	*p*‐value	*β* (95% CI)	*p*‐value	*β* (95% CI)	*p*‐value
12 weeks	Absolute weight loss, kg	1.01 (0.91, 1.10)	<0.0001*	0.20 (0.16, 0.24)	<0.0001*	−0.19 (−0.20, −0.18)	<0.0001*	−0.43 (−0.44, −0.41)	<0.0001*
	Relative weight loss, %	0.35 (0.25, 0.45)	<0.0001*	0.22 (0.18, 0.25)	<0.0001*	−0.07 (−0.08, −0.07)	<0.0001*	−0.47 (−0.48, −0.46)	<0.0001*
26 weeks	Absolute weight loss, kg	0.48 (0.30, 0.65)	<0.0001*	0.25 (0.18, 0.32)	<0.0001*	−0.29 (−0.30, −0.28)	<0.0001*	−0.66 (−0.69, −0.63)	<0.0001*
	Relative weight loss, %	−0.33 (−0.51, −0.15)	0.0003*	0.26 (0.19, 0.33)	<0.0001*	−0.15 (−0.16, −0.13)	<0.0001*	−0.70 (−0.73, −0.68)	<0.0001*
52 weeks	Absolute weight loss, kg	−0.10 (−0.36, 0.15)	0.43	0.15 (0.04, 0.25)	0.005	−0.35 (−0.38, −0.33)	<0.0001*	−0.69 (−0.74, −0.65)	<0.0001*
	Relative weight loss, %	−0.71 (−0.97, −0.44)	<0.0001*	0.11 (0.00, 0.21)	0.05	−0.24 (−0.26, −0.21)	<0.0001*	−0.74 (−0.79, −0.70)	<0.0001*

*Note*: Statistically significant results are marked with asterisks. The beta coefficients for ages are given as decades, and the coefficients for activity in the app are in tens of a percent for illustrative purposes.

**TABLE 5:** Multiple logistic regression analysis results.

Observation period	Dependent variable	Independent variables							
		Gender, female		Age, decades		Baseline BMI, kg/m^2^		In‐app activity, tens %	
		OR (95% CI)	*p*‐value	OR (95% CI)	*p*‐value	OR (95% CI)	*p*‐value	OR (95% CI)	*p*‐value
12 weeks	≥5% weight loss	0.88 (0.84, 0.93)	<0.0001*	0.88 (0.86, 0.90)	<0.0001*	1.03 (1.02, 1.03)	<0.0001*	1.22 (1.21, 1.23)	<0.0001*
	≥10% weight loss	0.76 (0.71, 0.82)	<0.0001*	0.80 (0.78, 0.83)	<0.0001*	1.04 (1.03, 1.04)	<0.0001*	1.18 (1.17, 1.20)	<0.0001*
26 weeks	≥5% weight loss	1.19 (1.12, 1.27)	<0.0001*	0.92 (0.90, 0.94)	<0.0001*	1.04 (1.03, 1.04)	<0.0001*	1.23 (1.22, 1.24)	<0.0001*
	≥10% weight loss	1.14 (1.05, 1.24)	0.00135	0.84 (0.82, 0.87)	<0.0001*	1.05 (1.04, 1.05)	<0.0001*	1.24 (1.23, 1.26)	<0.0001*
52 weeks	≥5% weight loss	1.33 (1.23, 1.44)	<0.0001*	0.91 (0.89, 0.94)	<0.0001*	1.05 (1.04, 1.06)	<0.0001*	1.20 (1.18, 1.21)	<0.0001*
	≥10% weight loss	1.41 (1.27, 1.56)	<0.0001*	0.84 (0.80, 0.87)	<0.0001*	1.06 (1.05, 1.07)	<0.0001*	1.24 (1.22, 1.26)	<0.0001*

*Note*: Statistically significant results are marked with asterisks. The odds ratios for ages are given as decades, and the coefficients for activity in the app are in tens of a percent for illustrative purposes.

All linear and logistic regression assumptions were checked and satisfied.

Using linear regression analysis, it was estimated that every additional 10% of in‐app activity (approximately, logging an action on 3 additional days within a 30 day period) increases absolute WL by 0.43 kg (95% CI 0.41, 0.44) after 12 weeks, by 0.66 kg (95% CI 0.63, 0.69) after 26 weeks, and by 0.69 kg (95% CI 0.65, 0.74) after 52 weeks. Interestingly, every additional 10 years of age was associated with a decreased absolute WL of 0.15–0.25 kg (Table 4).

According to the logistic regression analysis results, it was estimated that every additional 10 years of age decreases the chance of reaching minimal clinically significant WL by 1.08–1.12 times depending on the duration of the observation period. For the app usage, every extra 10% of in‐app activity (roughly, logging an action on 3 additional days within a 30 day period) increases the chance of reaching ≥5% WL by 1.2–1.23 times (Table 5).

## 4 DISCUSSION

This study demonstrates the effectiveness of engaging with the SIMPLE mobile application in achieving WL in adults with overweight and obesity in a real‐world context. App users within each activity segment (low, medium, high and maximal in‐app activity) were able to achieve clinically significant WL of ≥5%. Median WL and the proportion of users who achieved ≥5% WL increased as the frequency of app activity increased.

For those living with obesity, achieving ≥5% WL can help to improve various related comorbidities, such as reducing the risk of Type 2 diabetes, glycemic control in Type 2 diabetes, lipid profiles, and hepatic steatosis.^27^ This study found that 42.20% (95%CI: 42.95, 45.44) of active users lost ≥5% of their body weight at 52 weeks follow‐up. While the current study did not specifically capture data on health, these outcomes imply that SIMPLE could potentially serve as a promising intervention for improving overall health and supporting the national goal of reducing the number of Americans experiencing diet‐related diseases.^31^ Nonetheless, further research is necessary to validate this hypothesis.

Interestingly, these results align with existing literature,^32^ indicating that WL peaks at 6 months of follow‐up. The finding was that at 26 weeks median weight losses were greatest across all in‐app activity groups; while WL at 52 weeks remained robust, no additional WL occurred during this point with median WL decreasing slightly. This can be explained mainly by the fact that by the 52 weeks users experience WL plateau. These findings highlight the importance of the first 6 months in maximizing WL outcomes for those living with overweight and obesity before an anticipated deceleration. Mobile applications have the potential to optimize WL during this period due to their accessibility, interactive features, and leveraging novel tools such as AI chatbots and gamification.^33^

Demographic variations in WL were observed, with differences based on baseline BMI, age and sex. Notably, greater absolute and relative weight losses were observed among app users with a higher baseline BMI across all observation periods. App users of a higher age lost less weight than other groups at weeks 12 and 26, but at 52 weeks this association was no longer observed. Gender disparities were evident, with women generally experiencing less WL in the first 12 weeks than men, but were more successful than men at losing weight by 26 and 52 weeks. These findings are consistent with,^34^ whereby researchers found that women reported greater WL at 12 months of following TRE compared to men (−8.5 kg (95% CI: −10.4, −6.6) versus −7.6 kg (95% CI: −9.9, −5.3)). Altogether, this highlights the important sex‐specific differences in WL outcomes.

A key finding of this study is the effect of in‐app activity on WL outcomes; at each observation point, there was a dose‐response effect on WL when an increase in in‐app activity was observed. Notably, in the regression analysis, not only did this effect not weaken after the extended period between weeks 26 and 52, but it even increased slightly. This has also been seen elsewhere^35^ demonstrating the likely impact of this factor.

There were several strengths to this study. A key benefit of mobile applications is the large amount of data that can be collected. The high number of data points allowed sufficient statistical power to reach sound conclusions. The addition of regression analysis makes it possible to separate the influence of audience factors such as sex, age, and initial user weight from the variable of interest ‐ the overall level of activity in the app on WL. Regression analysis also allows us to draw clear conclusions about the potential quantitative impact of in‐app activity changes on WL. Another strength of this study is that it provides real‐world data, strengthening the ability to generalize results.

The study also has its limitations. Although real‐world data is advantageous in some ways, it also means that the data may be prone to some biases that would be minimized in an experimental design such as a randomized controlled trial. Furthermore, just as there are a large number of participants, many app users did not qualify to be a participant in the study due to having insufficient data to be included in the study as per the eligibility criteria. The criteria was defined to ensure sufficient data was available for meaningful analyses, similar practices are seen in studies evaluating other mobile applications supporting WL.^32^

As with similar studies, although some data were taken from connected devices, many of the weights were self‐reported, meaning weights could not always be validated. Due to the nature of the study, there was no alternative to the data collection method, and extensive consideration was paid to outlier removal to ensure the results were as accurate as possible.

While accessibility to traditional weight management services remains low for many, mobile health applications offer greater access to weight management support, with low marginal cost and broad access being a key lever to support management of the global obesity epidemic that continues to grow.^21^, ^36^

Real‐world evidence is valuable in evaluating the digital tools designed to support WL. Such data should be taken into account when considering the viability of such interventions.

Knowing that WL increases with higher app activity and that weight losses appear to be at their greatest at around 6 months, encouraging increased app usage and long‐term commitment is fundamental to WL achievement. Gamification and advanced behavior change techniques could encourage greater in‐app activity and therefore WL success, and should be considered in the development of mobile health applications for WL. For the SIMPLE app in particular, additional strategies such as gamification and advising specific and personalized actions for a user to complete each day could further increase engagement with the aim of promoting successful WL for more users.

It was outside the scope of the current study to evaluate the effects of different TRE schedules on WL, to estimate individual effects of different in‐app activities or their interaction with each other on WL, or to explore effects of different temporal properties of in‐app behavior on WL, for example, frequency of fasting or consistency of food logging; however, the authors suggest this should be evaluated in future research.

## 5 CONCLUSION

The SIMPLE mobile application for TRE is a successful WL tool. A clear dose‐response relationship exists between in‐app activity and mean WL or the proportion of users who achieve at least 5% and 10% WL. Elements that encourage app usage such as advanced behavior change techniques and gamification should be considered when developing mobile applications to support more users to achieve significant WL. Due to the proven success of mHealth offerings, mobile applications supporting WL should be considered as viable treatment options for patients, and real‐world observational studies should be considered when collecting evidence to make decisions regarding treatment for people living with overweight and obesity.

## AUTHOR CONTRIBUTIONS

Rosemary Huntriss and Dimitri Nikogosov conceptualized the study. Dimitri Nikogosov and Rodion Salimgaraev developed the study methodology, which was reviewed by John Powell and Krista A. Varady. Rodion Salimgaraev carried out the formal analysis and visualized the data. Rosemary Huntriss drafted the manuscript, and Krista A. Varady, John Powell, Rodion Salimgaraev and Dimitri Nikogosov reviewed and edited it.

## CONFLICT OF INTEREST STATEMENT

RH, DN and RS are employees of Simple.Life Apps Inc. The Simple.Life Apps Inc. administration had no role in the design of the study, in the collection, analyses, or interpretation of the data, in the writing of the manuscript, or in the decision to publish the results.

## Data Availability

The datasets generated and analyzed in this study are not publicly available due to the protection of confidential information of Simple.Life Apps Inc. business and Simple.Life Apps Inc. users.
